# Factors Associated With People’s Accessibility to Mental Healthcare Services in Ukraine: Focusing on Household Head Vulnerability

**DOI:** 10.3389/ijph.2023.1605890

**Published:** 2023-11-17

**Authors:** Min-Hee Heo, Eun-Mi Song, Hui-Won Jeon, Kyoung-Beom Kim, Jin-Won Noh

**Affiliations:** ^1^ Department of Health Administration, Yonsei University Graduate School, Wonju, Republic of Korea; ^2^ Department of Healthcare Management, College of Health Sciences, Youngsan University, Yangsan, Republic of Korea; ^3^ Department of International Healthcare Management, Catholic University of Daegu, Gyeongsan, Republic of Korea; ^4^ Division of Health Administration, College of Software and Digital Healthcare Convergence, Yonsei University, Wonju, Republic of Korea

**Keywords:** mental health, vulnerability, COVID-19, Ukraine, borderline

## Abstract

**Objectives:** This study examines the factors associated with access to mental healthcare services among people living in the government-controlled areas (GCAs) of Donetsk and Luhansk oblasts in Ukraine.

**Methods:** The 2020 Ukraine Multi-Sector Needs Assessment conducted by REACH was subjected to frequency analysis, percentage analysis, and binary logistic regression to confirm the factors associated with accessibility to mental healthcare services among Ukrainian household heads.

**Results:** Older household heads, heads with high accessibility to healthcare facilities, and those with low health expenditures were highly likely to have low access to mental healthcare services. Household heads’ awareness of household members’ medical assistance eligibility was significantly and positively associated with the former’s mental healthcare accessibility.

**Conclusion:** This study revealed the mental health vulnerability of people living in GCAs in Ukraine, in which the situation progresses from conflict to war. The need for mental healthcare, which is adversely affected by armed conflict, is expected to increase. Accordingly, further studies should clarify the demand for and methods to enhance mental healthcare services to ensure the timely provision of these services in the future.

## Introduction

Before the full-scale invasion by Russia, the fighting that lasted 8 years in eastern Ukraine caused heavy casualties, concerns regarding civilian protection, resilience capacity erosion, and critical infrastructure destruction in relevant areas [1, 2]. The armed conflict between Ukraine and pro-Russian separatist forces exposed civilians living in Donetsk and Luhansk oblasts (Donbas) on the eastern contact line to continuous and widespread conflict [1]. This ongoing conflict has caused young and working-age populations to leave the eastern Donbas region, resulting in the presence of a higher concentration of vulnerable populations in this area than in other regions of Ukraine [2].

As a result of this long-standing conflict, there were approximately 1.4 million internally displaced individuals in Ukraine as of July 2021 [3]. Furthermore, eastern Ukraine was divided into government-controlled areas (GCAs) and pro-Russian separatist rebel–controlled areas near the border. This division limited the movement of human and material resources for Ukrainian residents living on the border, which enhanced their vulnerability [1]. Further, displaced individuals mostly lived in unstable places, such as the houses of relatives or acquaintances, communal camps, and temporary facilities; were often unemployed; and experienced many restrictions regarding access to social support services [4, 5]. Vulnerable populations were highly likely to experience housing insecurity, unemployment, family separation, addiction problems, and mental disorders [6]. In addition, the outbreak of coronavirus disease 2019 (COVID-19) and adoption of restrictive disease control measures increased economic downturn and unemployment; these challenges can exacerbate the vulnerability of people living within 20 km of the line of contact in the GCAs of Donetsk and Luhansk oblasts [1]. The COVID-19 pandemic and armed conflict in Ukraine acted as stressors, enhancing the vulnerability of the affected people. The consecutive occurrence of these unprecedented events decreased the ability and capacity of Western countries to provide necessary humanitarian assistance [6]. A previous study indicated that vulnerable groups in developing countries with unstable social situations experience restrictions and difficulties in accessing mental healthcare services within national healthcare systems [7, 8]. Since socioeconomic constraints can be risk factors for depression and anxiety, the provision of mental health support to vulnerable Ukrainians must be emphasized [4, 5]. In Ukraine, the mental healthcare system focuses on inpatient care, with approximately 89% of the total mental health expenditure being allocated to inpatient services [9, 10]. Currently, the provision of mental health services in primary care and community settings is limited [9, 10]. Since 2014, the need for psychological assistance among the conflict-affected population has changed, and the prevalence of individuals experiencing the effects of violence has increased. Meanwhile, the public healthcare infrastructure largely remained unchanged or became more constrained [4]. The war imposed a substantial burden on the system and damaged or destroyed health facilities, such as secondary and tertiary mental health facilities. In the Donetsk and Luhansk regions, 77 of 350 and 26 of 250 healthcare facilities were damaged and destroyed, respectively [9]. According to an earlier study, the access to mental healthcare services in the Donetsk and Luhansk regions has been limited by travel and other constraints [9]. Furthermore, as of 2018, 83% of the people living in Donetsk and Luhansk lacked awareness of regional psychosocial support centers [4]. Therefore, this study examines the factors associated with the accessibility of mental healthcare services among Ukrainian residents within 20 km of the line of contact in the GCAs of Donetsk and Luhansk oblasts, and provides a basis of evidence to establish effective humanitarian support for vulnerable groups affected by the conflict.

## Methods

### Design and Data Collection

This study used the data from the 2020 Ukraine Multi-Sector Needs Assessment conducted by REACH for analysis [11]. REACH is a humanitarian initiative offering detailed data, timely information, and comprehensive analysis in crisis, disaster, and displacement contexts [11]. The 2020 Ukraine Multi-Sector Needs Assessment evaluated the proportions of households requiring humanitarian assistance and the severity of the requirements. These data were used to analyze the inter-sectoral severity of the needs of households within 20 km of the contact line. Large urban centers, such as Mariupol and Lysyschansk, were excluded from sampling to prevent overemphasis on the populations of urban centers, and to ensure the comprehensive coverage of rural and small urban regions. The data were collected through face-to-face interviews with the heads of households or household representatives. Subsequently, stratified sampling was performed across four areas: 5- or 20-km rural and urban regions. To obtain a representative sample of the general population, a 95% confidence level and 5% margin of error were established for each stratum. Further, the official population data provided by the State Statistics Service of Ukraine were used to weigh computerized random point selection within each region. Therefore, areas with high density were more likely to be selected for interviews, whereas uninhabited areas were less likely to be selected. Between 30 June and 15 August 2020, data were collected from 1,617 household heads and representatives in Donbas living in rural and urban regions between 0–5 and 5–20 km from the contact line [12]. Finally, 876 participants who responded to the study’s variables were included in the analysis.

### Variables

The dependent variable was accessibility to mental healthcare services, and relevant data were collected using the following question: “If any household members required mental health support, would they be able to access mental healthcare services?” This variable was categorized into yes or no. The study’s independent variables were the household head’s sociodemographic characteristics (gender, age, education level, marital status, income, the number of household members, house ownership, and region), vulnerability (displacement, disability, unemployment, veteran, single parent, three or more children, and chronic disease), and health-related characteristics (healthcare facility access, medical assistance awareness, and reduced health expenditures). Among the household head’s sociodemographic characteristics, the education variable was ascertained using the following question: “What is the highest level of education completed by the HH?” (HH refers to household head). The variable was categorized into secondary school and below or high school and above. The HH’s marital status variable was clarified by the question “what is the marital status of the HH?” and coded as single, married, or other (including widowed, divorced, or separated). Further, the income variable was assessed by the question “what is the total income of the HH per month?” and divided into 1–4 quantiles. The question “how many additional members does the HH have (total minus HH)?” ascertained the number of household members. Subsequently, house ownership was clarified by the question “do you own the house your household currently lives in?” and coded as yes or no. Further, the region variable was categorized into 5 km rural, 5 km urban, 20 km rural, and 20 km urban from the contact line. Regarding the HH’s vulnerability, the aforementioned variables were examined using the following question: “Does the HH have a vulnerability? If yes, what type?” Each type (HH with a disability, not including chronic illness; unemployed HH; HH who was a veteran of war; HH who was a single parent; HH with three or more children; and HH having a family with foster children) was coded as yes or no. Among health-related characteristics, the healthcare facility access variable was assessed by “how long does it take you to reach the nearest healthcare facility by walking?” and categorized into less than 3 h, more than 3 h, less than 15 min, less than 30 min, and less than 1 h. Subsequently, the medical assistance awareness variable was ascertained using the question “are you aware whether any member of your household is entitled to state-provided medications?” Finally, the variable for reduced health expenditures was obtained using the following question: “In the past 30 days, did anyone in your household have to engage in any of the following coping strategies to cope with reducing essential health expenditures (including drugs).”

### Statistical Analysis

Frequency and percentage analyses and binary logistic regression were performed to identify the factors associated with Ukrainians’ accessibility to mental healthcare services. Descriptive analyses summarized participants’ general, sociodemographic, vulnerability-related, and health-related characteristics. We performed multivariable binary logistic regression analysis to examine the factors related to the participants’ accessibility to mental healthcare services and reported the adjusted odds ratio (OR) with 95% confidence interval (CI) estimates. Further, a variance inflation factor (VIF) analysis was conducted to assess the correlations among variables. The average VIF value of 1.64 indicated the absence of multicollinearity for independent variables. All statistical analyses were performed using STATA/IC 15.1 (StataCorp LP, College Station, TX, United States). A significance level of 0.05 (two-tailed) was the threshold for statistical significance.

## Results

The analysis involved 876 representative samples (Table 1). Among HH, 22.26% (N = 195) were men and 77.74% (N = 681) were women. The HH’s mean age was 55.11 (min = 18, max = 95) years. In general, participants’ education level was low—83.56% (N = 732) had an education below the secondary school level. Most participants (N = 381, 43.49%) were married; 7.99% (N = 70) were single; and 48.52% (N = 425) were widowed, divorced, or separated (others). Further, 31.16%, 26.26%, 25.11%, and 17.47% of household income were 1, 4, 3, and 2 quantiles, respectively. The mean number of household members was 1.29 (min = 0, max = 8). Among the HH, 88.93% (N = 779) owned the houses in which they lived. Regarding region, 25.68% (N = 225) and 27.74% (N = 243) of the participants lived in rural regions located 20 and 5 km, respectively, from the contact line. Further, 24.43% (N = 214) and 22.15% (N = 194) of the participants lived in urban regions located 20 and 5 km, respectively, from the contact line. Most HH were found to have no disability (N = 792, 90.41%); only 9.59% of the HH recorded a disability. Further, 10% (N = 787) of the HH recorded a displacement status, and 12.10% (N = 106) of the HH were unemployed. Among the heads, 0.23% (N = 2) were veterans, 4.00% (N = 35) were single parents. It is noted that 3.08% (N = 27) of the HH had three or more children, and 46.92% (N = 411) had a chronic illness. Regarding health-related characteristics, approximately 31.51% of the HH had to walk less than 15 min to access a healthcare facility, 29.91% (N = 262) walked less than 30 min, 21.12% (N = 185) less than 1 h, 9.25% (N = 81) less than 3 h, and 8.22% (N = 72) more than 3 h. Moreover, 37.56% (N = 329) of the HH responded with reduced health expenditure, 30.25% (N = 265) had difficulty accessing the food market, and 41.78% (N = 366) could access mental healthcare services when their household members required them.

**TABLE 1:** General characteristic (Ukraine, 2020).

Variables		N	%
HH gender	Male	195	22.26
	Female	681	77.74
HH age^a^	(Min = 18, Max = 95)	(55.11 ± 15.63)	
HH education	≤Secondary	732	83.56
	≥High school	144	16.44
HH marital status	Single	70	7.99
	Married	381	43.49
	Others	425	48.52
HH income	1 Quantile	273	31.16
	2 Quantile	153	17.47
	3 Quantile	220	25.11
	4 Quantile	230	26.26
Number of HH members^a^	(Min = 0, Max = 8)	(1.29 ± 1.29)	
HH house ownership	No	97	11.07
	Yes	779	88.93
Region	20 km rural	225	25.68
	20 km urban	214	24.43
	5 km rural	243	27.74
	5 km urban	194	22.15
HH disabled	No	792	90.41
	Yes	84	9.59
HH displacement	No	787	89.84
	Yes	89	10.16
HH unemployed	No	770	87.90
	Yes	106	12.10
HH veteran	No	874	99.77
	Yes	2	0.23
HH single parent	No	841	96.00
	Yes	35	4.00
HH 3 more children	No	849	96.92
	Yes	27	3.08
HH chronic disease	No	465	53.08
	Yes	411	46.92
Healthcare facility access	Less than 15 min	276	31.51
	Less than 30 min	262	29.91
	Less than 1 h	185	21.12
	Less than 3 h	81	9.25
	More than 3 h	72	8.22
Medical assistance awareness	No	666	76.03
	Yes	210	23.97
Reduced health expenditure	No	547	62.44
	Yes	329	37.56
Food market access difficulty	No	611	69.75
	Yes	265	30.25
Mental health access	No	510	58.22
	Yes	366	41.78

^a^ Data are continuous variables (mean ± standard deviation), Abbreviation: HH, household head; h, hour.

To examine the factors related to mental healthcare services, multivariable binary logistic regression was performed on collected data (Table 2). An increase in HH’s age (OR = 0.97; *p* < 0.01; 95% CI = 0.96–0.99) was associated with a decrease in their access to mental healthcare services. HH with higher accessibility to healthcare facilities (less than 30 min OR = 0.62, *p* = 0.01, 95% CI = 0.43–0.89; less than 3 h OR = 0.45, *p* = 0.01, 95% CI = 0.26–0.80) and lower health expenditures (OR = 0.59; *p* < 0.01; 95% CI = 0.43–0.80) were more likely to have lower access to mental healthcare services than those with lower accessibility and higher expenditures. The awareness of household members’ medical assistance eligibility was significantly and positively associated with the accessibility to mental healthcare services (OR = 1.65; *p* = 0.01; 95% CI = 1.16–2.34).

**TABLE 2:** Factors associated with accessing for mental healthcare service (Reference group = no) (Ukraine, 2020).

Variables			OR	*p*-value	LL	UL
HH socio-economic factors	HH gender	Male	ref.			
		Female	1.30	0.16	0.90	1.87
	HH age	*Continuous*	0.97	<0.01	0.96	0.99
	HH education	≤Secondary	ref.			
		≥High school	1.00	0.99	0.68	1.48
	HH marital status	Single	ref.			
		Married	1.44	0.24	0.78	2.67
		Others	1.36	0.32	0.74	2.48
	HH income	1 Quantile	ref.			
		2 Quantile	1.16	0.52	0.74	1.82
		3 Quantile	1.26	0.27	0.84	1.88
		4 Quantile	1.17	0.48	0.76	1.81
	Number of HH members	*Continuous*	1.00	0.98	0.86	1.16
	HH house ownership	No	ref.			
		Yes	0.81	0.39	0.51	1.30
	Region	20 km rural	ref.			
		20 km urban	1.11	0.64	0.73	1.68
		5 km rural	1.15	0.48	0.78	1.71
		5 km urban	1.40	0.13	0.91	2.14
HH vulnerability factors	HH displacement	No	ref.			
		Yes	0.83	0.47	0.51	1.36
	HH disabled	No	ref.			
		Yes	0.82	0.42	0.50	1.34
	HH unemployed	No	ref.			
		Yes	0.97	0.89	0.60	1.56
	HH veteran	No	ref.			
		Yes	1.42	0.81	0.08	23.93
	HH single parent	No	ref.			
		Yes	0.65	0.29	0.29	1.44
	HH 3 more children	No	ref.			
		Yes	0.90	0.82	0.37	2.20
	HH chronic disease	No	ref.			
		Yes	1.13	0.52	0.79	1.62
Essential needs accessibility factors	Healthcare facility access	Less than 15 min	ref.			
		Less than 30 min	0.62	0.01	0.43	0.89
		Less than 1 h	0.69	0.07	0.46	1.03
		Less than 3 h	0.45	0.01	0.26	0.80
		More than 3 h	0.57	0.05	0.32	1.01
	Medical assistance awareness	No	ref.			
		Yes	1.65	0.01	1.16	2.34
	Reduced health expenditure	No	ref.			
		Yes	0.59	<0.01	0.43	0.80

*Hosmer-Lemeshow test chi^2^ value = 878.11; *p* = 0.21, VIF = 1.66, Abbreviation: HH, household head; h, hour; OR, odds ratio; LL, lower limit; UL, upper limit.

## Discussion

This study identified the factors associated with the accessibility of mental health services for Ukrainians living along the contact line. Results revealed that household members’ accessibility to mental healthcare services was significantly associated with the HH’s age, access to healthcare facilities, awareness of medical assistance eligibility, and decreased health expenditures. Although the result is not statistically significant, household representatives who experienced displacement had low accessibility to mental healthcare services since they were highly likely to experience the complex burdens of displacement, unemployment, and chronic illness and disabilities, all of which exacerbated poverty [8].

### Age of Household Heads

The household head’s age was negatively associated with accessibility to mental healthcare services. This finding is partially explained by an earlier study according to which household representatives of lower ages were more likely to have the capability to access healthcare facilities than those of higher ages [13]. Titus targeted regions having limitations in the physical environment and, thereby, a shortage of medical equipment or adequately trained medical personnel [13]. This study indicates that young, active, and productive household representatives are more likely to access the healthcare services provided by government hospitals than to perform self-care or accept traditional healthcare services [13]. This is because young people are more likely to travel long distances to access healthcare services than the elderly, and they can easily access information on service use.

### Reduction of Health Expenditure

The reduction in household health expenditure was negatively associated with the accessibility of mental healthcare services. This was because mental healthcare and medication payment burdens obstruct the reception of mental healthcare services [7]. This finding is attributed to the informal health expenses in Ukraine [14]. Although the government of Ukraine legally provides free medical services, individuals experience difficulty in paying high out-of-pocket expenses for healthcare services. Hence, the reduction in the medical expenditure of households burdened by such out-of-pocket expenses resulted in their poor access to mental health services. Furthermore, the Ukrainians living in contact lines experience more difficulty than those living elsewhere in accessing medical services due to the armed conflict-related increase in transportation, medicine, and drug costs, which cause an increase in out-of-pocket payments [4].

### Physical Accessibility of Healthcare Facilities

The physical accessibility of healthcare facilities was negatively associated with the accessibility of mental healthcare services for household members. This finding is similar to the result of an earlier study, which indicated that the shortage and unfair distribution of psychologists, psychotherapists, and social workers in Ukraine limited the accessibility of mental healthcare services [5]. The country’s mental healthcare services rely on centralized treatment through a few large psychiatric hospitals; however, these institutions are known to have a very poor service environment. Moreover, there is a lack of mental healthcare service personnel [5], which makes access to healthcare services time-consuming and lowers the utilization of healthcare facilities [13]. Prior studies have revealed that households with poor access to healthcare services are burdened by mental healthcare expenses. This finding supports the idea that low accessibility of mental healthcare services is caused by the burden of health expenses [5, 7]. Further, the closer the household is to a healthcare facility, the higher its utilization of healthcare services [13]. Previous study has shown that healthcare service use frequency depended on physical accessibility, travel and waiting times, and the utilization of an individual’s personal time to access the services [15].

### Awareness of Medical Assistance Eligibility

The HH who knew their household members’ eligibility to receive medical assistance were highly likely to access mental healthcare services; this is explained by the accessibility of health information. In low- and middle-income countries, mental health literacy is significantly associated with individual requests for help with mental health services [16]. Further, mental health literacy refers to the knowledge and beliefs that help recognize, manage, or prevent mental health disorders. This involves not only understanding mental health disorders but also requesting help to solve mental health problems, as required [17, 18]. This extends beyond the mere act of seeking help itself to the ability to find an effective service [16]. Prior studies report that vulnerable populations do not receive mental healthcare services because they are unaware of where the mental healthcare services are available [5]. In addition, people living in low-to middle-income countries often do not recognize or understand the importance of being mentally healthy and how mental health affects their lives. Even school staff, educators, and hospital health workers have limited mental health awareness and often do not know where to receive help [16]. Hence, individuals’ recognition of their own or household members’ medical assistance eligibility indicates their high understanding of health information or awareness of where to receive necessary services, that is, mental health literacy. Therefore, the household heads who know their household members’ medical assistance eligibility are highly likely to access necessary health information and, at the same time, have high access to mental healthcare services.

This study has several limitations. First, this study was conducted on household representatives in the Donbas region within 0–5 and 5–20 km from the contact line. Therefore, there are limitations in generalizing these findings to internally displaced person households. Further, both the GCAs and the non-government-controlled area of the Donbas region must be examined to obtain comprehensive results on household heads’ access to mental healthcare services. Second, the study had a cross-sectional design. Hence, there is a limitation to being a causal relationship. Third, due to data source limitations, household heads were included as household representatives. Further, the findings may be biased since they are based on individual perceptions and self-reported information. Finally, this study considered the household to be the unit of analysis and did not account for the household members’ individual characteristics.

Despite these limitations, this study highlighted the vulnerability of Ukrainians’ access to mental healthcare services in the current political situation, which is deteriorating from conflict to war. Since the collection of study data, one-third of Ukraine’s population has already fled their homes to avoid acts of aggression. However, the individuals internally displaced by war in the temporarily occupied parts of the Donetsk and Luhansk regions and the Crimean Peninsula receive only limited attention from media and politicians. Mental health and post-trauma rehabilitation services are important considerations in addressing the region’s high levels of vulnerability and ongoing conflict [3]. In Ukraine, mental healthcare services are mainly provided by governments; however, they involve only limited outreach support, and the residents living in contact line areas have low access to services due to a shortage of mental healthcare service providers [4]. In addition, the spread of disputes and conflicts to cities reduces overall access to medical facilities due to the destruction of infrastructure and private facilities [19]. For Ukrainians living in the contact line, access to mental healthcare services is further reduced by their locality’s distance from healthcare facilities and restrictions in physical movement. Currently, access to healthcare is an important issue faced by Ukrainians in conflict areas and the need for mental health, which has been adversely affected by armed conflict, is expected to increase in the future [4]. Therefore, the study’s findings are useful in the current context of increasing displacement of residents and disruption of mental health services. Future research should specify the demand for mental healthcare assistance and enhance service utilization to ensure the timely provision of mental healthcare services.
